# Major alteration of Lung Microbiome and the Host Reaction in critically ill COVID-19 Patients with high viral load

**DOI:** 10.21203/rs.3.rs-3952944/v1

**Published:** 2024-03-08

**Authors:** Ingrid G. Bustos, Rosana Wiscovitch-Russo, Harinder Singh, Benjamín L. Sievers, Michele Matsuoka, Marcelo Freire, Gene S. Tan, Mónica P. Cala, Jose L. Guerrero, Ignacio Martin-Loeches, Norberto Gonzalez-Juarbe, Luis Felipe Reyes

**Affiliations:** Universidad de la Sabana; J Craig Venter Institute; J Craig Venter Institute; J Craig Venter Institute; J Craig Venter Institute; J Craig Venter Institute; J Craig Venter Institute; MetCore- Metabolomics Core Facility, Universidad de Los Andes; MetCore- Metabolomics Core Facility, Universidad de Los Andes; St James’s Hospital; J Craig Venter Institute; Universidad de la Sabana

**Keywords:** Mechanical Ventilation, COVID-19, Microbiota, Cytokines, Metabolome, microbiota-virus-disease interactions

## Abstract

**Background:**

Patients with COVID-19 under invasive mechanical ventilation are at higher risk of developing ventilator-associated pneumonia (VAP), associated with increased healthcare costs, and unfavorable prognosis. The underlying mechanisms of this phenomenon have not been thoroughly dissected. Therefore, this study attempted to bridge this gap by performing a lung microbiota analysis and evaluating the host immune responses that could drive the development of VAP.

**Materials and methods:**

In this prospective cohort study, mechanically ventilated patients with confirmed SARS-CoV-2 infection were enrolled. Nasal swabs (NS), endotracheal aspirates (ETA), and blood samples were collected initially within 12 hours of intubation and again at 72 hours post-intubation. Plasma samples underwent cytokine and metabolomic analyses, while NS and ETA samples were sequenced for lung microbiome examination. The cohort was categorized based on the development of VAP. Data analysis was conducted using RStudio version 4.3.1.

**Results:**

In a study of 36 COVID-19 patients on mechanical ventilation, significant differences were found in the nasal and pulmonary microbiome, notably in *Staphylococcus* and *Enterobacteriaceae*, linked to VAP. Patients with VAP showed a higher SARS-CoV-2 viral load, elevated neutralizing antibodies, and reduced inflammatory cytokines, including IFN-δ, IL-1β, IL-12p70, IL-18, IL-6, TNF-α, and CCL4. Metabolomic analysis revealed changes in 22 metabolites in non-VAP patients and 27 in VAP patients, highlighting D-Maltose-Lactose, Histidinyl-Glycine, and various phosphatidylcholines, indicating a metabolic predisposition to VAP.

**Conclusions:**

This study reveals a critical link between respiratory microbiome alterations and ventilator-associated pneumonia in COVID-19 patients, with elevated SARS-CoV-2 levels and metabolic changes, providing novel insights into the underlying mechanisms of VAP with potential management and prevention implications.

## BACKGROUND

Since the emergence of the highly contagious Severe Acute Respiratory Syndrome Coronavirus-2 (SARS-CoV-2) in 2019, the COVID-19 pandemic has rapidly spread worldwide, leading to profound global health and economic consequences (1). The World Health Organization (WHO) has reported a staggering 770 million cases and nearly 7 million deaths globally by July 2023 (2, 3). Notably, 5–12% of patients progress to severe or critical stages, necessitating invasive mechanical ventilation (IMV) and significantly increasing mortality rates (4–6). However, IMV often triggers complications, including secondary infections, which can worsen clinical outcomes and extend stays in intensive care units (ICUs) and hospitals (4, 7). Critically ill COVID-19 patients often experience bacterial superinfections, further complicating their condition.

In the intensive care setting, individuals with severe COVID-19 pneumonia show a marked propensity for respiratory superinfections, with mechanical ventilation-associated pneumonia (VAP) being especially prevalent. This tendency is thought to be associated with SARS-CoV-2 virus-induced alterations of the pulmonary microbiota (8, 9). The occurrence of VAP and other superinfections may be attributed to the invasion of new pathogens or bacterial strains, which diversify from primary SARS-CoV-2 infection (10, 11). Data suggests that at least 32% of these patients will develop bacterial superinfections, increasing morbidity and mortality rates (12, 13). However, the exact prevalence and impact of initial bacterial superinfections on progression to VAP in patients with severe COVID-19 pneumonia are not yet fully understood (11). The dynamics of the pulmonary microbiome are thought to play an integral role in initiating and shaping the course of superinfections and influencing patient response to treatment. Understanding these interactions is essential to improve therapeutic strategies and patient outcomes in severe cases of COVID-19 (14).

The lungs harbor a diverse microbiome comprising approximately 100 different bacteria, viruses, and fungi (15, 16). This complex microbiome is crucial in maintaining immune balance and significantly influences the severity and duration of respiratory infections, such as SARS-CoV-2 (1, 17). The intricate interplay between the commensal microbiota and the immune system is vital for regulating immune responses, with microbiota-derived metabolites mediating these interactions. Additionally, metabolic changes have been observed, but their connection to bacterial superinfections in severe COVID-19 patients remains unclear (1, 18, 19). Changes in the microbiome-immune system interplay due to host-microbiome dysbiosis may lead to dysregulated immune responses and conditions like systemic inflammation (20, 21). It is crucial to comprehend these interactions. This study explores how SARS-CoV-2 affects the lung microbiome in critically ill COVID-19 patients on mechanical ventilation. We analyze the microbiome, metabolites, and host immune response to understand better the underlying mechanisms responsible for VAP in 36 mechanically ventilated COVID-19 patients.

## MATERIALS AND METHODS

This prospective cohort study was conducted at Clinica Universidad de La Sabana in Chia, Colombia, between January 2021 and July 2021, including all critically ill COVID-19 patients requiring invasive mechanical ventilation admitted to the ICU. The attending physicians prospectively gathered data by reviewing medical records and laboratory results in the platform for data storage REDCap every time the patient was screened and selected. Nasal swabs (NS), Endotracheal aspirates (ETA), and blood samples were collected in the initial 12 hours following intubation, and a follow-up was conducted 72 hours post-intubation. Then, we performed microbiological analysis, cytokines, and metabolomic characterization. The Institutional Review Board (IRB) of Clinica Universidad de La Sabana approved the study, and all patients provided informed consent to participate (CUS-20190903). All methods and research procedures were performed in accordance with the local and international regulations for good clinical practices in clinical research and did not change the clinical treatment of the patients participating in the study as per local clinical guidelines.

### Study population

Patients diagnosed with COVID-19 and required ICU admission and invasive mechanical ventilation within 12 hours of hospital admission for more than 72 hours were included in this study (Table 1). The severity of COVID-19 was classified based on WHO guidelines, and critical illness was identified in patients who needed invasive mechanical ventilation, extracorporeal membrane oxygenation (ECMO), or suffered from end-organ dysfunction (22). We excluded pregnant patients who had been invasively ventilated in another hospital. Patients who had been administered more than two doses of antibiotics before intubation, those who had IMV for over 24 hours before the sample collection, and patients who had a documented coinfection within 48 hours of admission were also excluded. Demographic data, comorbidities, symptoms, physiological variables, systemic complications, and laboratory reports from the first 24 hours of admission were recorded and monitored every 48 hours until the patient was extubated. We retrospectively reviewed the data from medical records at the time of hospital discharge to ensure the accuracy of the recorded information uploaded to the REDCap platform hosted at the Universidad de La Sabana (Plataforma REDCap - Universidad de La Sabana [Internet]. Universidad de La Sabana; [2023]. Disponible en: https://redcap.unisabana.edu.co/).

### Recollection and sample processing

ETA and NS samples were meticulously collected following established protocols employing sterile saline (0.9%). Immediately post-collection, these samples were frozen at −80°C segregated into distinct aliquots for future sequencing and metabolomics analyses. Prior to these analyses, the samples underwent thawing and thorough mixing to eradicate any particulate matter. Concurrently, blood samples were obtained through an intravenous catheter, utilizing 5- or 10-mL Becton Dickinson Vacutainers (red top tubes), and then centrifuged at 1,970 × g for 10 minutes. Subsequently, the supernatant was methodically apportioned into aliquots and preserved at −80°C for ensuing processing. To maintain consistency in handling and storage, thereby minimizing potential contamination or degradation risks, the research team collected all blood samples, ensuring rigorous standardization and enhancing the accuracy of the analyses. Samples were obtained from eligible patients on invasive mechanical ventilation within the initial 24 hours (day 0) and subsequently on days 3, 5, and 7 or the day of diagnosis of mechanical VAP.

### Diagnosis Criteria for VAP

The diagnosis of VAP was based on current clinical guidelines published by the Infectious Diseases Society of America and the American Thoracic Society (IDSA/ATS) for the management and diagnosis of VAP (23). Diagnostic criteria included patients on mechanical ventilation for at least 72 h, a new or progressive radiographic infiltrate, and at least two of the following symptoms: fever (body temperature > 38 °C), purulent tracheal secretions, or leukocytosis or leukopenia (leukocyte count > 10,000/μL or < 4,000/μL, respectively). Patients were included in the VAP category only if, after being intubated to the ICU for 48 hours or more, they had at least one respiratory pathogen isolated from their ETA (>106 CFU) or bronchoalveolar lavage (>104 CFU) that is known to cause pneumonia.

### DNA extraction

DNA isolation was performed using the DNeasy^®^ Blood & Tissue Kit from QIAGEN, a commercially available kit. Initially, a 500 μL sample obtained from either a ETA or NS was centrifuged at 6,750 × g for 10 minutes at room temperature. Subsequently, the supernatant was removed, and the pellet was resuspended in 200 μL of PBS. The isolation process followed the manufacturer’s instructions. The quality and concentration of DNA samples were assessed using the NanoDrop^™^ One instrument.

### 16S r RNA amplification and sequencing

Amplification and sequencing of the V4 region of the 16S rRNA gene were performed using primers 515–533F forward (GTGCCAGCMGCCGCGGTAA) and 806–787R reverse (GGACTACHVGGGTWTCTAAT) with 8-bp barcode and Illumina adaptor (24). The polymerase chain reaction (PCR) was carried out using approximately 100 ng of gDNA per sample and Thermo Fisher Platinum Taq DNA Polymerase (Cat# 10966–026, Life Technologies, Carlsbad, CA). The amplification conditions were as follows: 94°C for 5 min, 94°C for 30 s, 55°C for 30 s, 72°C for 30 s for 35 cycles, 72°C for 7 min. The libraries were purified using QIAquick PCR purification kit to remove primer-dimers and short reads (<100bp) and quantified using Qubit 1X dsDNA HS Assay (Cat# 28106, QIAGEN, Hilden, Germany). The libraries were normalized, and fragment size was examined using a sensitivity DNA Kit (Cat# 5067–4,626, Agilent, Santa Clara, CA). The library pool was sequenced using the Illumina MiSeq system as instructed by the manufacturer (Cat# MS-102– 3,003, Illumina Inc., La Jolla, USA). A low amount of environmental and reagent contamination was detected in most of the PCR-negative controls (**Supplemental Fig. 1**).

The bioinformatic analysis involved demultiplexing and generating fastq files using CASAVA v1.8.2 (Illumina Inc., La Jolla, CA). The fastq files were filtered using KneadData to remove low-quality reads (<Q30), end trimming, and contamination from host mitochondrial sequences (25). An in-house bioinformatic pipeline supported by Mothur (26) and Uparse (27) with SILVA 16S rRNA database (version 123) was used to assign Operational taxonomical units (OTUs) at 97% sequence similarity (28). The relative abundance and diversity plots were generated using R packages phyloseq and ggplot 2 (29).

### Cytokines/Chemokines/growth factor measures

The analysis of various protein targets was conducted utilizing the Invitrogen^™^ multiplexed immunoassay panel, specifically, the Cytokine/Chemokine/Growth Factor 45-Plex Human ProcartaPlex^™^ Panel 1 (Cat #EPX450–12171-901, ThermoFisher Scientific, Vienna, Austria), in accordance with the manufacturer’s instructions. Serum samples were processed using a compatible Luminex 200 instrument (Luminex Corporation, Austin, Texas, USA), utilizing lot# 313189–002 for bead mixes, detection antibody mixes, and standard mixes, all prepared as per manufacturer’s instructions. To ensure accuracy, the combined standards were diluted fourfold and run in duplicate alongside two blanks containing assay buffer only. Prior to analysis, samples were thawed on ice, subjected to centrifugation at 1,000 × g for 10 minutes, and the supernatant was analyzed without further dilution.

Following data collection, quality control measures were implemented according to a specified protocol (30). All samples had a bead count exceeding 100, with a minimum requirement of 30 beads. After analysis with the Luminex, Mean Fluorescence Intensity (MFI) was provided and was transformed to Net MFI after subtracting the background from the blank wells. Using the ProcartaPlex Analysis App (ThermoFisher Scientific, Vienna, Austria), concentration values were generated via transformation of Net MFI based on the standard curves for each analyte, as we previously reported for saliva (31) and serum(32). Target concentrations were adjusted to standardized values. Values labeled OOR< or OOR> were adjusted to match the lowest (Standard 7) or highest (Standard 1) limit of detection, respectively. The ranges of concentrations (pg/ml) for each target are included in the Supplementary Materials. After this transformation, all values were log10-transformed. The samples from the VAP-COVID and NO VAP-COVID groups were analyzed separately for each target using Mann-Whitney tests. Results were visually represented through box graphs displaying mean values and standard deviations.

### Untargeted Metabolomic Analysis

The untargeted metabolomic investigation employed two methods: RP-LC-QTOF-MS and HILIC-LC-QTOF-MS. Sample preparation involved adding cold methanol (3:1 ratio) to plasma, vortexing for 5 minutes, and centrifugation at 7,310 × g for 10 minutes at 4°C. The analysis integrated an Agilent 1260 Infinity LC System with a 6545 Q-TOF LC/MS system from Agilent Technologies in Waldbronn, Germany. A 2 μL sample was injected into a ZORBAX Eclipse Plus C18 column (2.1 × 50 mm, 1.8 μm particle size) at 60°C. Mobile phases were 0.1% formic acid in water (A) and 0.1% formic acid in acetonitrile (B) with a flow rate of 0.6 mL/min.

The HILIC-LC-QTOF-MS analysis involved injecting 5 μL of the sample into an Infinity Lab Poroshell HILIC-Z column (2.1 × 100 mm, 1.9 μm particle size) maintained at a constant temperature of 30°C. The mobile phases comprised 10% (200 mM ammonium format in Milli-Q water, pH 3) with 90% water (phase A) and 10% (200 mM ammonium format in water, pH 3) mixed with 90% acetonitrile (phase B). The flow rate remained constant at 0.6 mL/min, employing a gradient elution program. Data acquisition was conducted in negative electrospray ionization mode (ESI-), covering a mass-to-charge ratio spectrum from 50 to 1100 m/z.

### Statistical analysis

Statistical analysis was performed using GraphPad Prism 9 software and R statistical framework (version 4.3.1). Initially, we used the Shapiro–Wilk test to assess the data distribution rigorously. Descriptive statistics were systematically applied to summarize the data set, encompassing the mean with standard error and the median coupled with the interquartile range (IQR). Chi-square tests were judiciously applied for categorical variables to compare patient characteristics between distinct groups, while independent t-tests were utilized for continuous variables.

We estimated microbial diversity using the sophisticated vegan package implemented within the R environment. Alpha diversity was meticulously evaluated employing both Shannon and Chao1 indices. The significance of differences in alpha diversity between groups was determined by applying Wilcoxon’s rank sum test or the Mann–Whitney U-test. The selection of these tests was contingent on whether the data were paired or unpaired. Beta diversity was quantified using the Bray-Curtis dissimilarity index and the weighted UniFrac distance. Principal Coordinate Analysis (PCoA) was conducted to assess beta diversity across varying groups. This involved using permutational multivariate analysis of variance (PERMANOVA), incorporating 9,999 permutations facilitated by the adonis2 function in the Vegan R package (v2.6–4).

To analyze the differences between groups, ratios were evaluated employing Fisher’s exact probability test. Furthermore, correlations between clinical indicators and the lung microbiota were analyzed using Spearman Correlation Analysis. Throughout, a *p-value* threshold of less than 0.05 was adhered to, denoting statistical significance in all analytical determinations. For metabolomics comparative analysis, two-sample t-tests were applied, and the mean of groups was used to calculate the fold-change values.

## RESULTS

**Figure 1:**
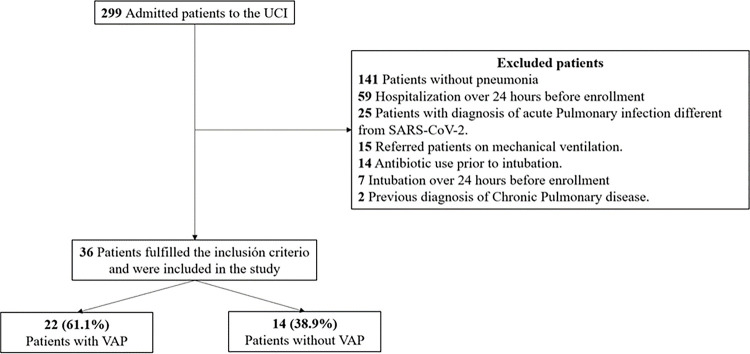
Study Flow Chart. Flow diagram for the study showing the number of patients included in the analysis.

106 samples were collected from 36 COVID-19 patients undergoing mechanical ventilation in the ICU. This collection comprised 36 NS and 70 ETA samples (Fig. 1). Utilizing 16S RNA gene sequencing, the study delved into investigating the microbial composition within the respiratory tracts of these patients. The cohort was characterized by its diversity, encompassing individuals who either developed or did not develop VAP, thereby permitting a thorough evaluation of microbial diversity in severe COVID-19 cases. Demographic data, clinical characteristics, and laboratory test results are systematically presented in Table 1.

The median age of the cohort was 56 years, with an IQR of 49.7 to 64.2 years. A noteworthy finding was the prevalent administration of antimicrobials among these patients; 52.7% (19/36) received antimicrobials at ICU admission. When comparing patients with and without VAP, those admitted exhibited a markedly prolonged median duration of ICU length of stay (VAP: 15.0 days [IQR: 19.0–24.0] vs. No VAP: 6.0 days [IQR: 3.0–11.0], p < 0.01), extended length of hospital stays (VAP: 29.0 days [IQR: 12.0–48.5] vs. No VAP: 11.0 days [IQR: 4.0–18.0], p < 0.01), and an increased length of invasive mechanical ventilation (VAP: 9.0 days [IQR: 7.0–14.0] vs. No VAP: 3.0 days [IQR: 2.0–5.0], p < 0.01). These findings underscore the significant disparities between patients who developed VAP and those who did not.

### COVID-19 Patients with VAP and without VAP show differential nasal microbiome abundance changes upon ICU admission.

**Figure 2:**
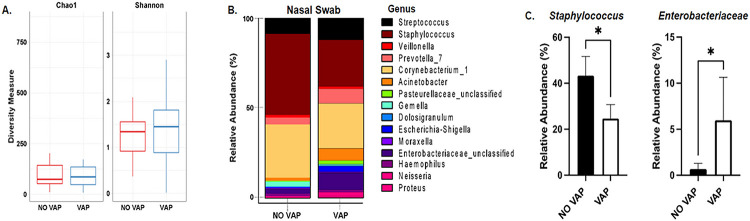
Nasal swabs of patients with COVID-19 that develop ventilator-associated pneumonia showed differential abundance of Staphylococcus and Enterobacteriaceae. **A.** Alpha diversity of nasal microbiome from COVID-19 patients that developed VAP or did not (NO VAP). **B.** Percent relative abundance of the top 15 most abundant microbes in the nasal cavity. **C.** Relative abundance bar graphs of Staphylococcus and Enterobacteriaceae genus. Student’s t-test was used to calculate the *p-value*. Asterisks denote the level of significance observed: * = p ≤ 0.05; ** = p ≤ 0.01; *** = p ≤ 0.001.

We first used Chao and Shannon diversity measures to test for differences in microbial abundance changes between the groups. Although no significant alterations were discerned among the groups in the overall microbial composition (Fig. 2A), further investigations were conducted to probe for specific abundance shifts among the predominantly present organisms within the samples. This in-depth analysis was designed to unearth subtle discrepancies potentially obscured in the broader comparative framework, yielding a more intricate and nuanced understanding of microbial dynamics. These showed significant differences between the VAP and NO VAP groups (Fig. 2B), specifically in bacteria from the genus *Staphylococcus* and *Enterobacteriaceae* (Fig. 2C). *Staphylococci* are Gram-positive bacteria that are common skin, pulmonary, and oral commensals, and members of this genus can also be pathobionts (33, 34). In contrast, members of the genus *Enterobacteriaceae* are part of a family of Gram-negative bacteria that includes pathogens such as *Klebsiella, Enterobacter, Citrobacter, Salmonella, Escherichia, Shigella, Proteus, Serratia* among others (35). These data suggest a possible shift in nasal colonizers that may predispose the patient to VAP from the members of the *Enterobacteriaceae* bacterial genus.

### Endotracheal aspirates from COVID-19 patients who develop VAP have a reduction of Staphylococcus and increased Gram-negative bacterial pathogens.

**Figure 3.**
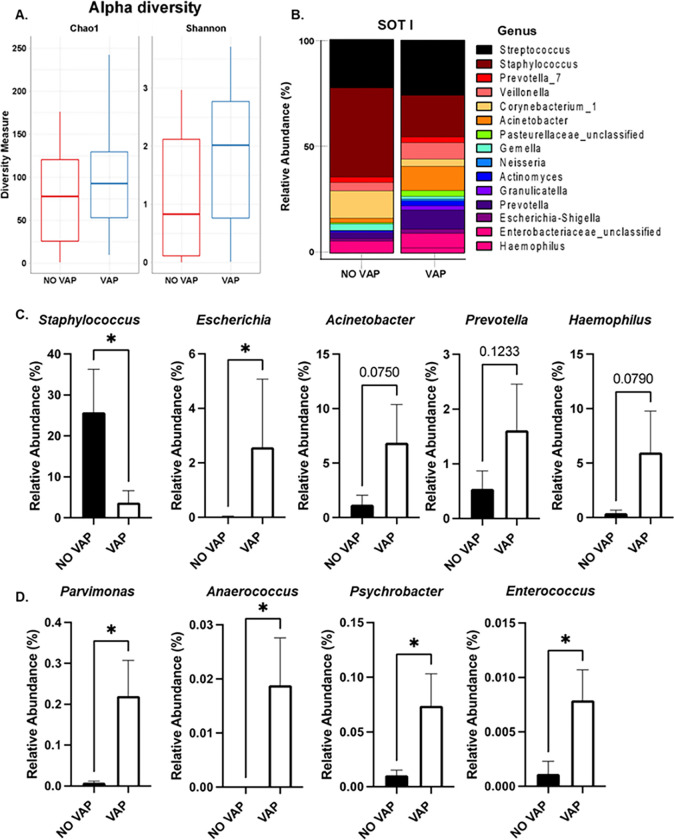
Endotracheal aspiration of patients with COVID-19 shows differential abundance of pulmonary microbiome upon mechanical ventilation. **A.** Alpha diversity of pulmonary microbiome from COVID-19 patients that developed VAP or did not (NO VAP). **B.** Percent of relative abundance of the top 15 most abundant microbes in the lungs. **C.** Relative abundance bar graphs of *Staphylococcus, Escherichia, Acinetobacter, Prevotella*, and *Haemophilus* genus. **D.** Relative abundance bar graphs of *Parvimonas*, *Anaerococcus, Psychrobacter*, and *Enterococcus* genus. Student’s t-test was used to calculate the *p-value*. Asterisks denote the level of significance observed: * = p ≤ 0.05; ** = p ≤ 0.01; *** = p ≤ 0.001.

To further assess pulmonary microbiome changes in the cohort, ETA samples were collected from patients upon intubation and at a follow-up time point (72 hours). At baseline, the Chao test did not show changes in total microbial richness. However, an increase in the Shannon index showed that richness and evenness were higher in the VAP group (Fig. 3A). Changes in abundance of the top 15 microbial genus showed drastic differences between the group who developed VAP and those who did not (Fig. 3B). Statistical analysis of the most abundant genus revealed a reduction in *Staphylococcus* and an increase in members of the *Enterobacteriaceae* group (Fig. 2). More precisely, a significant alteration in the abundance of *Escherichia* was observed, alongside a notable trend approaching significance in *Acinetobacter*.

Furthermore, increases in *Prevotella* and *Haemophilus* were also detected. However, these changes did not reach statistical significance (Fig. 3C). We also tested for significant changes in less abundant bacteria as they may influence the growth of pathogens by alteration of the local microenvironment. Of note, we observed a significant increase in the abundance of *Parvimonas, Anaerococcus, Psychrobacter*, and *Enterococcus* (Fig. 3D). Upon testing microbial changes in a follow-up time point, similar trends in microbial abundance were observed (**Supplemental Fig. 2**). Taken together, these results suggest that patients who develop VAP have an altered nasal and pulmonary microbiome that may predispose them to this severe form of disease.

### A higher abundance of SARS-CoV-2 in serum correlates with dynamic changes in nasal and pulmonary microbiome in VAP patients.

**Figure 4:**
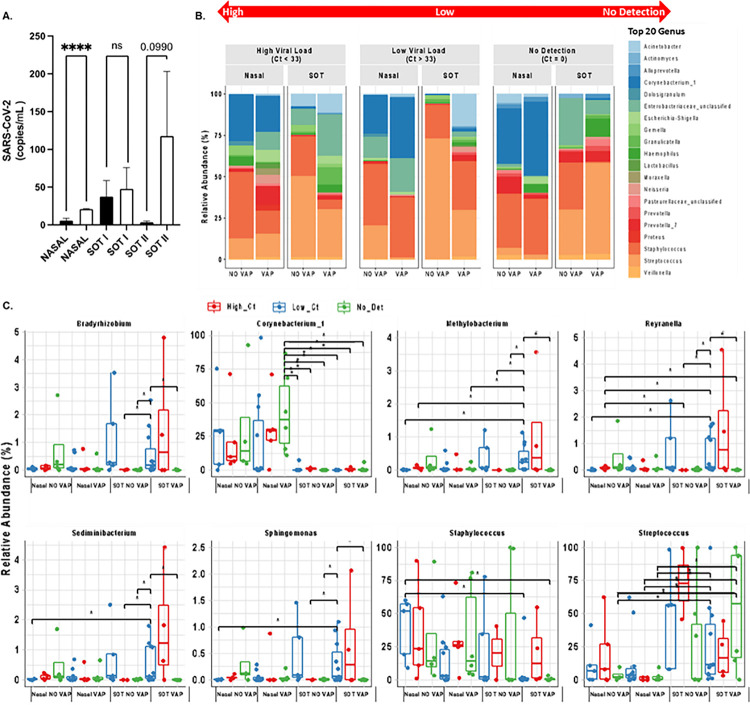
Differential abundance of SARS-CoV-2 modulates the nasal and lung microbiome. **A.** Log copies per mL of SARS-CoV-2 were tested via quantitative RT-PCR. **B.** Percentage of relative abundance of the top 20 most abundant microbes in the nasal cavity and the lungs. Student’s t-test was used to calculate the *p-value*. Asterisks denote the level of significance observed: * = p ≤ 0.05; ** = p ≤ 0.01; *** = p ≤ 0.001.

To determine the potential association between serum viral load and shifts in nasal and pulmonary microbiota, the research quantified levels of SARS-CoV-2 in the nasopharynx and lungs of the cohort at key intervals: upon hospital admission, during mechanical ventilation and at a subsequent follow-up. The findings indicated that patients who developed VAP exhibited higher Log copies/mL of SARS-CoV-2 at admission (the initial assessment point), as determined via quantitative real time polymerase chain reaction (RT-PCR) (Fig. 4A). Notably, significant variations in bacterial abundance were observed among patients with differing viral titers of SARS-CoV-2, compared to those without detectable virus at the time of sample collection, in both nasal and lung samples (Fig. 4B-C).

In nasal samples, the group with a higher viral load displayed a reduction in *Corynebacterium* and *Staphylococcus* and an increase in *Proteus, Enterobacteriaceae*, and *Escherichia-Shigella* (Fig. 4B). Conversely, the group with a lower viral load demonstrated an increase in *Corynebacterium* and *Enterobacteriaceae*, and a decrease in *Streptococcus* (Fig. 4B). In cases with no detectable SARS-CoV-2 in nasal samples, a reduction in *Acinetobacter* and *Prevotella*, and an increase in *Corynebacterium* and *Haemophilus* were noted (Fig. 4B).

Regarding pulmonary samples, the high viral load group also exhibited a decrease in *Corynebacterium* and *Staphylococcus* and an increase in *Acinetobacter, Enterobacteriaceae*, and *Haemophilus* (Fig. 4B). In contrast, the lower viral load group showed an increase in *Acinetobacter, Neisseria*, and *Haemophilus*, and a decrease in *Streptococcus* (Fig. 4B). For pulmonary samples with undetectable SARS-CoV-2, a reduction in *Enterobacteriaceae* and *Staphylococcus* and an elevation in *Streptococcus* and *Haemophilus* were observed (Fig. 4B).

When analyzing all samples collectively, statistical differences in the relative abundance of *Bradyrhizobium*, *Methylobacterium, Reyranella, Sediminibacterium*, and *Sphingomonas* were also noted (**Supplemental Fig. 3**). In summary, the data suggest that viral titers are linked with a diminution in commensal bacteria and an escalation in Gram-negative pathogenic bacteria, potentially contributing to the development of VAP in patients under mechanical ventilation.

### COVID-19 patients who developed VAP showed increased SARS-CoV-2 neutralizing antibodies and decreased inflammatory cytokines and chemokines.

**Figure 5:**
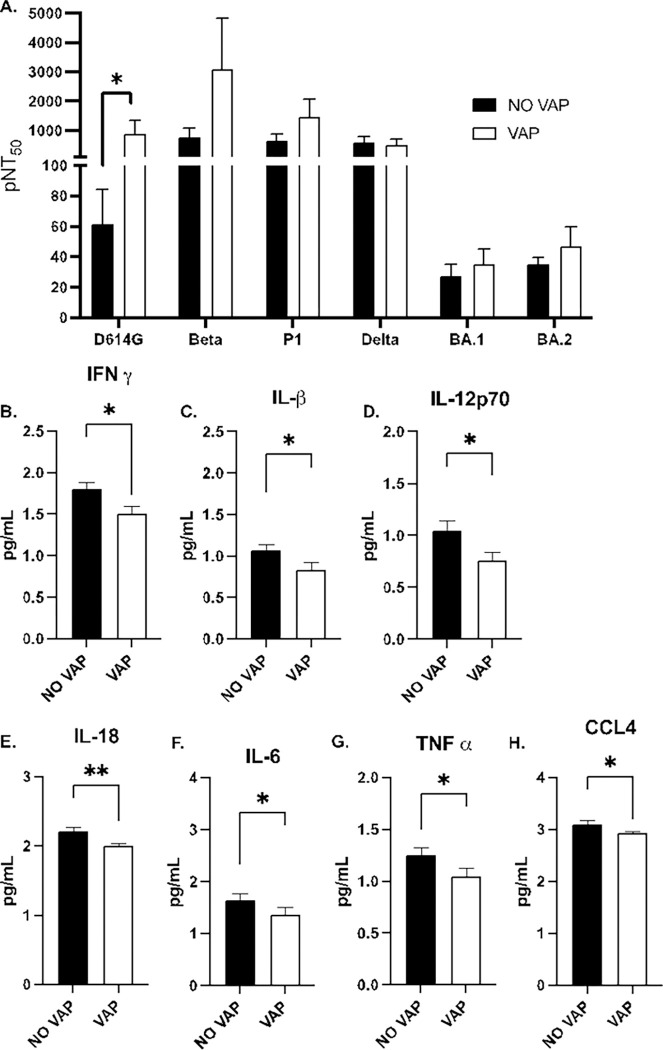
Plasma-neutralizing antibody titers and inflammatory effectors are differentially regulated during COVID-19 associated VAP. **A.** pNT50 values against the four SARS-CoV-2 pseudo-viral variants Beta, Gamma, Delta, and Omicron BA.1 and BA.2 and the control early 2020 strain with the D614 mutation. They were measured in samples collected from COVID-19 patients who developed VAP and those who did not (NO VAP). **B.** Cytokine and chemokine changes in serum (pg per mL). Mann-Whitney test was used to calculate the *p-value*. Asterisks denote the level of significance observed: * = p ≤ 0.05; ** = p ≤ 0.01; *** = p ≤ 0.001.

Spike-specific neutralizing antibodies are widely acknowledged as key indicators of the immune response against viruses and bacteria. Given that all patients in the study were diagnosed with COVID-19, the research aimed to ascertain if notable differences existed in neutralization titers between the groups with and without VAP. The analysis revealed no significant disparities in the capacity to neutralize pseudoviruses from variants of concern, namely Beta, Gamma, Delta, and Omicron subvariants BA.1 and BA.2. However, a marked elevation in the neutralization of D614 (closest to the original strain from 2019–2020) was discerned in the VAP group (Fig. 5A).

Additionally, plasma samples were procured from the 36 COVID-19 patients to quantify cytokines and chemokines. A significant reduction was observed in IFN-δ (*p*=0.01; Fig. 5B), IL-1β (*p*=0.04; Fig. 5C), IL-12p70 (*p*=0.01; Fig. 5D), IL-18 (*p*=<0.01; Fig. 5E), IL-6 (*p*=0.04; Fig. 5F), TNF-α (*p*=0.04; Fig. 5G), and CCL4 (MIP-1) (*p*=0.0479; Fig. 5H) in patients who developed VAP compared to those who did not. These findings suggest that in the VAP group, at the time of ICU admission, both a pronounced efficacy of neutralizing antibody activity and a decrease in inflammatory cytokines and chemokines are implicated in an antiviral response that might diminish host effectiveness against bacterial infections. Furthermore, the data indicates that the development of VAP was not linked to any specific viral variant of concern.

### Differential metabolomic changes occur in COVID-19 patients who develop VAP.

**Figure 6:**
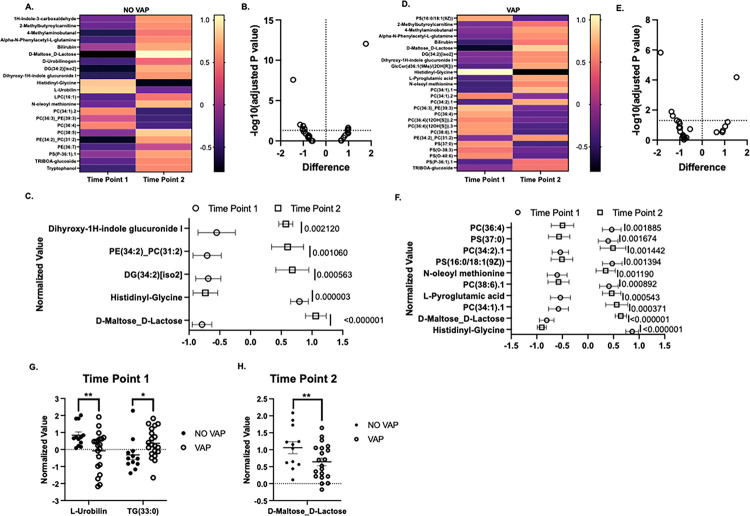
Metabolomic changes in serum are observed during COVID-19-associated VAP. **A.** Heat map of the significantly changed metabolites when comparing NO VAP group at baseline and follow-up time point. **B.** Volcano plot for significant metabolites that pass the threshold of 1.3 −log10 (adjusted p-value). C. Most changed metabolites from the NO VAP group at baseline and follow-up time points. **D.** Heat map of the significant metabolite changes when comparing the baseline and follow-up time points of the VAP group. **E.** Volcano plot for significant metabolites that pass the threshold of 1.3 −log10 (adjusted p-value). **F.** Most changed metabolites from the VAP group at baseline and follow-up time points. **G.** Changes in L-Urobilin and TG normalized values (33:0) in VAP vs. No VAP at time point 1. **H.** Changes in normalized values of D-Maltose_D-Lactose in VAP vs. No VAP at time point 2. Student’s t-test was used to calculate the *p-value*. Asterisks denote the level of significance observed: * = p ≤ 0.05; ** = p ≤ 0.01; *** = p ≤ 0.001

To test the effects of metabolic changes in COVID-19 patients who do or do not develop VAP, we carried out global metabolomics in plasma at two different time points, upon mechanical ventilation (baseline) and after 72 hours (follow-up). In patients without VAP, we observed significant changes in 22 metabolites (Fig 6A). After setting up a threshold of −log10 of 1.3 of the adjusted p value (Fig 6B). The most notably altered metabolites in this group were identified as D-Maltose-Lactose, Histidinyl-Glycine, Diacylglycerol with 34 carbons and 2 double bonds (DG 34:2), Phosphatidylethanolamine and Phosphatidylcholine combination with 34 carbons and 2 double bonds in total (PE 34:2_PC 31:2), Dihydroxy-1H-indole, and Glucuronide I (Figure 6C). For the patients who developed VAP, we observed that 27 metabolites had significant changes between the two-time points (Figure 6D). Upon evaluating the adjusted p-values of metabolites surpassing the −log10 threshold of 1.3 (Figure 6E), it was discerned that the most significantly altered metabolites in the VAP cohort included Histidinyl-Glycine, a combination of Maltose and Lactose, Phosphatidylcholine with a total of 34 carbons and 1 double bond (PC 34:1), Pyroglutamic acid, Phosphatidylcholine with a total of 38 carbons and 6 double bonds (PC 38:6), a derivative of oleoyl methionine, Phosphatidylserine with 34 carbons and 1 double bond (PS 16:0/18:1), Phosphatidylcholine with 34 carbons and 2 double bonds (PC 34:2), Phosphatidylserine with 37 carbons (PS 37:0), and Phosphatidylcholine with 36 carbons and 4 double bonds (PC 36:4) (Figure 6F).

When contrasting the NO VAP and VAP groups at the baseline, only Urobilin and Triglyceride with a total of 33 carbons differed significantly between the groups (Figure 6G). At the follow-up, the comparison revealed a significant difference in the levels of the maltose-lactose combination (Figure 6H). Urobilin and maltose-lactose exhibited higher concentrations in the NO VAP group compared to the VAP group. The fluctuations in Urobilin might be linked to hepatic involvement, either as a direct consequence of the disease or due to certain medications administered. Alterations in the maltose-lactose combination are associated with shifts in the gut microbiome and a decrease in Short-Chain Fatty Acid (SCFA) producing bacteria (PMC7002114). SCFAs are crucial in modulating bacterial pathogen load and the level of inflammation (PMC8370681). Notably, Triglyceride with 33 carbons, also referred to as TG 17:0/8:0/8:0, was significantly elevated in patients who developed VAP (Figure 6G). This triglyceride variant has been implicated in inflammation modulation and lipid metabolism, corroborating the findings presented in Figure 5. Collectively, this data suggests that changes in specific metabolites might serve as a mechanism predisposing COVID-19 patients to VAP.

## DISCUSSION

The microbiomes of the upper respiratory tract (URT) and lower respiratory tract (LRT) play a pivotal role in maintaining respiratory health by exerting influence over the severity of respiratory viruses, such as SARS-CoV-2, and potentially shaping acute immune responses (36–38). Given that the URT serves as the primary entry point for the COVID-19 virus, it is imperative to gain a thorough understanding of how the URT microbiome may impact the severity and outcomes of COVID-19 (39, 40). In our study, we observed notable disparities in the abundance of nasal microbiomes between COVID-19 patients who developed VAP and those who did not. While there were no significant alterations in the overall microbial diversity, discernible differences emerged in specific microbial abundances, particularly within the bacterial genera *Staphylococcus* and *Enterobacteriaceae*. These findings suggest a potential shift in nasal microbial colonization patterns that may contribute to an elevated susceptibility to VAP in afflicted patients. This observation aligns with prior studies, which have demonstrated overlap in the composition of the upper respiratory tract and lung microbiomes, indicating a role in overall pulmonary health (41). Furthermore, it is consistent with existing literature implicating opportunistic pathogens, such as Staphylococcus, in the severity of respiratory viral infections (42).

In ETA samples collected at the time of intubation and during a follow-up assessment, we observed a heightened microbial diversity and evenness in patients with VAP. Shifts in microbial abundance patterns indicated a reduction in *Staphylococcus* and an increase in *Enterobacteriaceae*, particularly *Escherichia spp*. These findings align with reports from other studies where *S. aureus* and *E. coli* were identified as the most common causative microorganisms of VAP in COVID-19 patients (43–45). Additionally, less abundant bacteria, typically undetectable through conventional culture methods for VAP diagnosis, such as *Parvimonas, Anaerococcus, Psychrobacter, Prevotella*, and *Enterococcus*, also exhibited significant increases in patients who developed VAP. Similar trends were observed in the follow-up samples, indicating altered nasal and pulmonary microbiomes in VAP patients. Furthermore, an initial higher abundance of *Streptococcus* was observed in the baseline samples, followed by a subsequent decrease in the follow-up samples, irrespective of their classification as VAP or non-VAP cases.

Dysbiosis in the microbiome can foster an inflammatory milieu that facilitates the invasion and replication of the coronavirus, thereby constituting a risk factor for disease severity (46, 47). Our study conducted a comparative analysis of immune responses in COVID-19 patients with and without VAP. We discovered that spike-specific neutralizing antibodies exhibited similar efficacy against various SARS-CoV-2 variants in both groups, except for a notable increase in neutralization against the D614 variant within the VAP group. Furthermore, we assessed cytokine levels and observed diminished concentrations of pivotal cytokines in the VAP cohort, indicative of a subdued inflammatory response. These findings imply that individuals with VAP mount a robust neutralizing antibody response while concurrently exhibiting a reduced inflammatory cytokine profile (IFN-δ (*p*=0.01), IL-1β (*p*=0.04), IL-12p70 (*p*=0.01), IL-18 (*p*=<0.01), IL-6 (*p*=0.04), TNF-α (*p*=0.04), and CCL4 (MIP-1) (*p*=0.0479) upon admission to the ICU. This phenomenon may influence their immune defenses against bacterial infections, although no specific viral variant association with VAP was established.

This metabolomic analysis of COVID-19 patients unveils distinct metabolic profiles, sharply differentiating those with VAP from those without. The study identified 47 metabolites across various chemical classes and metabolic pathways, significantly altering phospholipid, sphingolipid, and glutathione metabolism in VAP patients. These changes, affecting cell membrane integrity and oxidative stress, could play a crucial role in VAP’s pathogenesis, potentially enhancing bacterial adhesion and destabilizing immune responses (48). Additionally, imbalances in metabolites critical for glutathione and sphingolipid synthesis may exacerbate these effects, underlining their importance in VAP’s complex pathophysiology. Conversely, patients without VAP exhibited variations in glycerophospholipids, glucuronides, and indole compounds, suggesting robust immune and metabolic responses. Glycerophospholipids indicate an optimal cellular membrane composition, vital for immune efficiency and cellular integrity, while variations in dihydroxyl, a glucuronide and indole compound, highlight its role in liver detoxification (49). Elevated urobilin levels in non-VAP patients point to preserved hepatic function, crucial for processing heme byproducts, contrasting with lower levels in VAP patients, possibly indicating liver impairment due to disease severity or medication side effects.

A primary limitation of this study is its relatively small sample size. Nevertheless, a comprehensive and multifaceted methodology was adopted to enhance the understanding of pulmonary microbiota dynamics in COVID-19 patients, focusing on developing secondary infections. These findings underscore the complex impact of COVID-19 and emphasize the critical importance of a holistic research approach. Such an approach deepens the understanding of the disease’s complexities and opens new avenues for prevention and treatment strategies, thereby making substantial contributions to the advancement of COVID-19 and respiratory infection management and patient care.

## CONCLUSION

Our study elucidates the intricate interplay between the respiratory microbiota and COVID-19, emphasizing the significance of microbiome variations in patients with and without VAP. Employing advanced 16S RNA gene sequencing on samples from COVID-19 patients, we identified distinct microbial compositions correlated with disease severity. These findings reveal a critical link between microbial dysbiosis and the severity of COVID-19, suggesting that specific alterations in microbiota, alongside patient immunology, and metabolites, may influence both viral and bacterial pathogenesis. Despite the constraints of a small sample size, this research substantially contributes to the COVID-19 field, advocating for a holistic approach to treatment strategies and patient care. It also foregrounds the necessity for further exploration into the microbiome’s role in respiratory diseases, particularly in severe viral infections, highlighting the imperative for a comprehensive understanding of these complex interactions to enhance patient outcomes amid ongoing global health challenges.

## Figures and Tables

**Table 1.** Demographic information, clinical characteristics, and laboratory test indices of patients were stratified into two groups: those with VAP and those without VAP

Characteristic	All n = 36	VAP baseline n = 24	No-VAP baseline n = 12	p-value	VAP follow up n = 25	No VAP follow-up n =11	p-Value
**Demographic**							
Male. N (%)	22 (61)	14 (58)	8 (66)	0.90	14 (56)	7 (63)	1
Age. Median (IQR)	56.0 (49.7–64.2)	57.5 (50.0–64.2)	54.5 (47.7–61.5)	0.51	57.5 (50.0–64.2)	54.0(46.5–64.0)	0.54
**Comorbid conditions. N (%)**							
Anemia	1 (2.8)	0 (0)	1 (8.3)	0.71	0 (0)	1 (9.1)	0.68
Cancer	1 (2.8)	1 (4.2)	0 (0)	1	1 (4.0)	0 (0)	1
Diabetes mellitus	2 (5.6)	1 (4.2)	1 (8.3)	1	1 (4.0)	1 (9.1)	1
Coronary disease	1 (2.8)	1 (4.2)	0 (0)	1	1 (4.0)	0 (0)	1
COPD	1 (2.8)	1(4.2)	0 (0)	1	1 (4.0)	0 (0)	1
Arterial hypertension	12 (33.3)	10 (41.7)	2 (16.7)	0.26	10 (40.0)	2 (18.2)	0.32
Obesity	9 (25.0)	6 (25.0)	3 (25.0)	1	6 (24.0)	3 (27.3)	1
No background	18 (50.0)	12 (50.0)	6 (50.0)	1	12 (48.0)	5 (45.5)	1
**Physiological variables during the first 24 hours of admission. Median (IQR)**							
Heart rate. BPM	93.5 (77.2–106.0)	85.5 (73.5–103.5)	98.0 (90.7–125.5)	0.07	83.5 (63.7–99.0)	82.0 (67.0–87.0)	1
Respiratory rate. RPM	24.0 (20.0–30.0)	24.0 (20.0–25.7)	24.5 (20.0–40.0)	0.31	24.0 (20.0–24.2)	24.0 (20.0–24.0)	0.91
Temperature. °C	36.6 (36.5–36.9)	36.5 (36.5–36.9)	36.9 (36.4–37.0)	0.28	36.9 (36.6–37.5)	37.0 (37.0–37.4)	0.22
SBP mmHg	118.0 (105.0–134.2)	119.5 (107.5–133.2)	115.0 (101.5–137.8)	0.76	128.0 (117.8–144.5)	126.0 (105.5–146.5)	0.63
DBP mmHg	65.5 (58.7–73.2)	66.0 (59.5–73.2)	65.5 (57.0–69.5)	0.67	66.0 (60.2–72.5)	71.0 (65.0–78.5)	0.27
PAM. mmHg	84.3 (75.2–89.5)	85.5 (75.2–89.5)	83.1 (73.7–88.5)	0.62	86.8 (81.2–98.1)	90.6 (81.5–94.8)	0.80
SPO2. (%)	85.5 (80.7–90.0)	84.0 (80.0–90.0)	90.0 (81.0–93.7)	0.12	90.0 (87.5–92.0)	90.0 (84.5–92.0)	0.97
Glasgow Coma Scale	8.5 (6.0–15.0)	8.0 (6.0–15.0)	14.0 (6.0–15.0)	0.68	6.0 (6.0–6.2)	6.0 (6.0–7.0)	0.96
**Laboratory variables at admission. Median (IQR)**							
WBC, cell × 103	10.7 (8.10–14.0)	9.9 (7.1–13.0)	13.0 (11.0–16.5)	0.06	9.4 (7.9–13.5)	12.9 (9.4–16.9)	0.39
Neutrophiles, (%)	85.5 (80.7–90.2)	86.5 (81.0–90.5)	82.5 (79.7–89.5)	0.34	84.0 (81.7–90.2)	89.0 (80.0–92.0)	0.48
Hemoglobin, g/dL	14.8 (13.8–16.0)	14.8 (13.9–16.0)	14.8 (13.5–16.0)	0.91	12.5 (11.2–14.0)	11.6 (11.1–12.0)	0.13
Platelet, cell × 103	230.0 (180.0–28.0)	226.5 (150.0–252.5)	275.0 (205.8–356.8)	**0.02**	200.0 (167.5–252.5)	243.0 (189.30–315.0)	0.31
Creatinine, mg/dL	0.9 (0.8–1.1)	1.0 (0.9–1.4)	0.8 (0.7–0.9)	**0.04**	1.3 (0.9–2.2)	0.8 (0.7–1.4)	0.10
BUN, mg/dL	20.0 (15.0–26.0)	22.5 (16.2–29.7)	15.0 (13.0–18.0)	**0.01**	32.5 (22.7–45.0)	24.0 (18.0–41.5)	0.27
Blood glucose, mg/dL	142.0 (124.5–185.0)	145.0 (129.2–180.0)	142.0 (121.0–210.0)	0.98	150.0 (130.0–180.0)	150.0 (150.0–170.0)	0.71
Sodium, mEq/L	139.0 (136.8–140.2)	139.0 (137.5–140.5)	139.0 (136.0–139.0)	0.78	144.0 (139.8–145.2)	145.0 (143.0–147.5)	0.18
Potassium, mEq/L	4.3 (4.0–4.5)	4.3 (4.1–4.6)	4.2 (4.0–4.5)	0.50	4.8 (4.2–5.2)	4.2 (4.1–4.8)	0.22
pH	7.31 (7.20–7.41)	7.33 (7.20–7.41)	7.25 (7.17–7.38)	0.62	7.34 (7.20–7.42)	7.42 (7.30–7.45)	0.24
PCO2, mmHg	46.0 (34.0–58.2)	45.0 (34.0–53.2)	57.5 (34.0–65.2)	0.38	46.5 (43.0–54.5)	46.0 (44.5–53.0)	0.78
PaO2, mmHg	68.5 (59.0–75.2)	64.5 (59.0–73.0)	79.0 (65.5–89.7)	**0.04**	64.0 (60.2–66.0)	59.0 (57.0–67.5)	0.83
FiO2	70.0 (45.0–90.0)	80.0 (45.0–91.2)	52.5 (43.7–82.5)	0.27	40.0 (39.2–48.2)	40.0 (35.0–52.5)	0.66
HCO3, mmol/L	24.0 (20.1–26.0)	23.5 (19.7–26.0)	24.0 (21.7–27.0)	0.55	26.0 (21.0–29.2)	30.0 (27.5–31.0)	0.13
Acid lactic, mmol/L	1.4 (1.1–2.1)	1.5 (1.1–2.1)	1.3 (1.1–1.7)	0.46	1.3 (1.0–1.8)	1.1 (0.9–1.2)	0.31
**Outcomes. Median (IQR)**							
Length of stay in ICU, days (IQR)	8.0 (4.0–14.0)	15.0 (9.0–24.0)	6.0 (3.0–11.0)	**<0.01**	15.0 (9.0–24.0)	10.0 (6.0–13.5)	**<0.01**
Length of stay in the hospital, days (IQR)	13.0 (7.0–29.0)	29.0 (12.0–48.5)	11.0 (4.0–18.0)	**<0.01**	29 (12.0–48.5)	15.0 (10.5–22.5)	**0.02**
Intubation time, days (IQR)	5.0 (3.0–9.0)	9.0 (7.0–14.0)	3.0 (2.0–5.0)	**<0.01**	9.0 (7.0–14.0)	6.0 (5.0–8.5)	**<0.01**
Hospital Mortality (%)	30 (83.3)	19 (79.2)	11 (91.7)	0.63	19 (76.0)	10 (90.9)	0.70
Mortality 28d (%)	30 (83.3)	19 (79.2)	11 (91.7)	0.63	19 (76.0)	10 (90.9)	0.70
Mortality 90d (%)	31 (86.1)	20 (83.3)	11 (91.7)	0.86	20 (80.0)	10 (90.9)	0.94
**Scores. Median (IQR)**							
SOFA	8.0 (7.0–9.0)	8.0 (7.2–9.0)	8.0 (6.0–9.0)	**0.01**	9.0 (7.0–10.0)	9.0 (7.0–10.0)	0.85
APACHE	15.0 (10.0–20.0)	17.0 (14.0–23.2)	14.0 (9.0–19.2)	**0.01**	17.0 (14.0–19.0)	14.0 (11.0–20.0)	0.10
CPIS	2.0 (10.0–4.0)	2.0 (1.0–3.0)	2.0 (1.0–5.0)	0.30	3.0 (1.0–4.0)	2.0 (1.0–2.30)	**<0.01**

**Abbreviations:** VAP: Ventilator-Associated Pneumonia, N: Number, IQR: Interquartile Range, BPM: Beats Per Minute, SPO2: Peripheral Oxygen Saturation, COPD: Chronic Obstructive Pulmonary Disease, WBC: White Blood Cells, PCO2: Partial Pressure of Carbon Dioxide, PaO2: Partial Pressure of Oxygen, FiO2: Fraction of Inspired Oxygen, HCO3: Bicarbonate, ICU: Intensive Care Unit, SOFA: Sequential Organ Failure Assessment, APACHE: Acute Physiology and Chronic Health Evaluation, CPIS: Clinical Pulmonary Infection Score.

## Data Availability

The datasets generated and/or analyzed during the current study are available in the National Center for Biotechnology Information (NCBI) repository, project PRJNA1079780, submission ID SUB14266176 (https://dataview.ncbi.nlm.nih.gov/object/PRJNA1079780?reviewer=sn0018pk13mthrvc792dknogk8). All the other clinical data is available by request to the corresponding authors.

## Abbreviations

VAP: Ventilator-associated pneumonia
NS: Nasal swabs
ETA: Endotracheal aspirates
SARS-CoV-2: Severe Acute Respiratory Syndrome Coronavirus-2
WHO: World Health Organization
IMV: Invasive mechanical ventilation
ICU: Intensive care unit
ICUs: Intensive care units
IRB: Institutional Review Board
IDSA/ATS: Infectious Diseases Society of America and the American Thoracic Society
CFU: Colony forming units
OTU: Operational taxonomical
MFI: Mean Fluorescence Intensity
IQR: Interquartile range
PCoA: Principal Coordinate Analysis
PERMANOVA: Permutational multivariate analysis of variance
RT-PCR: Real time polymerase chain reaction
SCFA: Short-Chain Fatty Acid
URT: Upper respiratory tract
LRT: Lower respiratory tract
ECMO: Extracorporeal membrane oxygenation
PCR: Polymerase chain reaction
